# Genome-wide association study identifies loci for the general combining ability of Pythium root and stalk rot caused by *Pythium arrhenomanes* in maize (*Zea mays* L.)

**DOI:** 10.1270/jsbbs.25069

**Published:** 2026-06-16

**Authors:** Shohei Mitsuhashi

**Affiliations:** 1 NARO Institute of Livestock and Grassland Science, Senbonmatsu, Nasushiobara, Tochigi 329-2793, Japan

**Keywords:** best linear unbiased prediction, general combining ability, genome-wide association study, marker-assisted selection, Pythium root and stalk rot, *Zea mays* L.

## Abstract

*Pythium arrhenomanes* Drechsler is a destructive soil-borne pathogen threatening forage maize (*Zea mays* L.) production worldwide, leading to substantial economic losses due to root and stalk rot (RSR). Developing resistant varieties is the most sustainable and effective management strategy. However, studies analyzing general combining ability (GCA) for disease resistance using genome-wide association study (GWAS) are lacking. This study investigated the genetic architecture of resistance to Pythium RSR using 107 maize inbred lines using GCA for disease resistance as a phenotypic trait, addressing instability of disease expression in inbred lines and targeting additive genetic variance crucial for F_1_ hybrid breeding. A GWAS was conducted using 49,187 high-quality single-nucleotide polymorphism (SNP) markers. Five SNPs were significantly associated with resistance GCA that did not form linkage disequilibrium blocks in their vicinity. One SNP on chromosome 9 conferred a beneficial reduction in GCA, indicating an increase in resistance. Four of the five SNPs exhibited an apparent effect and were polymorphic between the resistant ‘Na71’ and susceptible ‘Ho112’ inbred lines, indicating their direct applicability in applied breeding. Overall, these loci are strong candidates for marker-assisted selection, providing a direct route to accelerate the development of forage maize hybrids with enhanced disease resistance and agronomic performance.

## Introduction

Maize (*Zea mays* L.) is a major global crop fundamental to food, feed, and bioenergy systems, with worldwide production reaching approximately 1.2 billion tons (FAO 2023). Forage maize, cultivated for whole-plant silage or grain usage, is an indispensable component of the livestock industry, providing high-energy feed for dairy and beef cattle. However, the productivity and quality of forage maize are affected by a range of soil-borne diseases. Pathogens belonging to genera such as *Pythium*, *Fusarium*, and *Rhizoctonia* cause seed decay, seedling damping-off, and root or root and stalk rot (RSR), which collectively decrease plant stand, biomass, and grain yield (Bickel and Koehler 2021, Harvey *et al.* 2008). Economic losses attributed to seedling blights and root rots caused by *Pythium* species are severe; in the United States and Ontario alone, these diseases are estimated to result in annual losses amounting to tens of millions of dollars, potentially reaching over $350 million in recent years (Bickel and Koehler 2021).

*Pythium arrhenomanes* is a particularly destructive and widespread pathogen that causes root rot, making its management challenging (Deep and Lipps 1996, Reyes-Tena *et al.* 2018). In Japan, particularly south of the Kanto region, Pythium RSR affects the roots and stalks during the maturing stage (Tsukiboshi *et al.* 2014, Fig. 1). Fungicides such as metalaxyl-M provide inconsistent chemical control and may lead to the development of pathogen resistance (Porter *et al.* 2009). Additionally, crop rotations are less effective due to the pathogen’s extensive host range, which includes other major crops as well as common ornamental plants (Wu *et al.* 2020a). This enables the inoculum to remain and accumulate in the soil over consecutive seasons. Therefore, developing varieties with robust genetic resistance is the most economically viable, environmentally sustainable, and effective long-term strategy for controlling Pythium RSR. Although considerable progress has been made in understanding resistance to other maize pathogens such as *Fusarium* (Guo *et al.* 2020), *Puccinia polysora* (Sun *et al.* 2021), and *Pythium aristosporum* (Hou *et al.* 2023), the genetic basis of resistance to *P. arrhenomanes* remains comparatively under-explored.

Host resistance to necrotrophic pathogens such as *Pythium* is typically a quantitative trait. Quantitative disease resistance is controlled by multiple genes or quantitative trait loci (QTLs), each contributing a small-to-moderate effect (Hou *et al.* 2023, Lin *et al.* 2020). This polygenic inheritance makes resistance more durable against evolving pathogen populations but also more complex to manipulate in breeding programs. Genome-wide association study (GWAS) can identify specific genomic regions, and even individual single-nucleotide polymorphism (SNPs), that are significantly linked to the trait of interest, making it an effective tool for gene discovery and marker development (Zuffo *et al.* 2022).

A primary challenge in QTL analysis or GWAS is the acquisition of reliable phenotypic data. As indicated in our previous studies, Pythium RSR symptoms can be unstable and often absent when assessed directly on inbred lines (Mitsuhashi 2024, Mitsuhashi *et al.* 2015). Therefore, rather than measuring an unreliable trait in inbred lines, the general combining ability (GCA) of the inbred lines was used, calculated based on the more stable and agronomically relevant performance of their F_1_ hybrid progenies across multiple environments (Mitsuhashi 2024, Mitsuhashi and Tamaki 2022).

By conducting GWAS on these GCA values, the additive genetic variance that breeders aim to accumulate for improved hybrid performance can be directly targeted. This approach effectively addresses limitations in phenotypic analysis as well as facilitates the isolation of resistance genes as traits with high heritability that are suitable for selection.

Single sequence repeat analysis of GCA for yield, plant height, and ear height in maize has been conducted (Zheng *et al.* 2021). Additionally, studies have reported GWAS analyses of GCA in rice (Chen *et al.* 2019, Eltahawy *et al.* 2020). However, studies analyzing GCA for disease resistance as GWAS phenotypes are lacking.

Here, this study aimed to 1) identify SNP loci significantly associated with resistance to Pythium RSR by conducting a GCA-based GWAS, and 2) evaluate the identified loci for their immediate applicability in a practical marker-assisted selection (MAS) program, thereby providing effective tools to accelerate the development of disease-resistant maize.

## Materials and Methods

### Plant materials and field tests

In this study, a diversity panel consisting of 107 maize inbred lines was used. This panel was compiled to indicate a broad genetic background appropriate for maize breeding programs in Japan and other temperate regions. The pre-calculated GCA values for each of the 107 inbred lines were used, as previously determined by Mitsuhashi and Tamaki (2022, 2024) based on the RSR infection frequencies of F_1_ varieties in field tests. Although GCA values were calculated for 202 inbred lines, only the 107 inbred lines with available genotype data described below were finally used.

Field experiments were conducted from 2014 to 2024, at the ILGS, NARO, Nasushiobara, Tochigi, Japan (36°55ʹ04ʺN, 139°56ʹ29ʺE; 320 m above mean sea level). A maize–Sudan grass (*Sorghum sudanese* [Piper] Stapf.) annual rotation was implemented for fields. In the early spring of each year (except for 2022 when the experiment was suspended), 50 metric tons ha^–1^ of manure, 600 or 700 kg ha^–1^ of fertilizer (containing 17 or 14% each of N, P_2_O_5_ and K_2_O) and 60 kg ha^–1^ insecticide (Diazinon granule) were applied before sowing. Some herbicides (6.0 L ha^–1^ of Alachlor emulsion and 2.0 L ha^–1^ of Atrazine wettable powder) were also applied after sowing.

All field tests were conducted in a randomized complete block with two to four replicates. All entries were grown in single row plots (2.4 or 3.6 m in length, and 0.75 m apart), each of which consisted of 13 or 19 plants. When each F_1_ hybrid reached the yellow-ripe stage (about 50 days after silking), the plants were cut at about 5 cm above the ground, and the degree of rotting on the cut surface of the stalks was recorded. Pythium RSR resistance was evaluated by the infection frequency as the percentage of plants whose scores were two or more (Mitsuhashi *et al.* 2015). As the fields were presumed to have a high level of Pythium RSR pathogen contamination, F_1_ hybrids were evaluated under natural infection. Sowing and observation dates were different for each year or environment. Table 1 shows the scale and outline of field tests.

### GCA calculation

To predict the breeding values (GCAs) of individual inbred lines, the BLUP mixed model matrix equation in maize by Mitsuhashi (2024) was used, which incorporates both GCA and special combining ability (SCA) into the model:


$$y=X\beta +Z_{g}a_{1}+Z_{s}a_{2}+\varepsilon$$ (1)

where y represents the phenotypic value, defined as the infection frequencies of Pythium RSR in the field based on 946 F_1_ data from 2014 to 2024; X is a design matrix that indicates which F_1_ combination is tested in which field experiments; β is an unknown vector for the environmental values of each experiment; $a_{1}$ and $a_{2}$ are vectors for the GCA and SCA of each inbred line; $Z_{g}$ is a design matrix that indicates which inbred lines are the parents of each F_1_ combination; $Z_{s}$ is a design matrix that indicates the combination of F_1_ for each data; and ε is a residual effect. In each component of the design matrix $Z_{g}$, the directionality of the crosses is excluded. The inbred lines used for the F_1_ combination are defined as 1, while the inbred lines not used are defined as 0. This model did not consider the effects of epistasis.

The solution of the matrix equation Eqn. (1) was based on previous studies:


$$(\begin{array}{c} X^{\prime }R^{-1}y\\ {Z_{g}}^{\prime }R^{-1}y\\ {Z_{s}}^{\prime }R^{-1}y \end{array})=\left(\begin{array}{c} X^{\prime }R^{-1}X\\ {Z_{g}}^{\prime }R^{-1}X\\ {Z_{s}}^{\prime }R^{-1}X \end{array}\;\begin{array}{c} X^{\prime }R^{-1}Z_{g}\\ {Z_{g}}^{\prime }R^{-1}Z_{g}+G_{1}^{-1}\\ {Z_{s}}^{\prime }R^{-1}Z_{g} \end{array}\;\begin{array}{c} X^{\prime }R^{-1}Z_{s}\\ {Z_{g}}^{\prime }R^{-1}Z_{s}\\ {Z_{s}}^{\prime }R^{-1}Z_{s}+G_{2}^{-1} \end{array}\right)\;\left(\begin{array}{c} \hat{\beta }\\ \hat{a_{1}}\\ \hat{a_{2}} \end{array}\right)$$ (2)

where $X^{\prime }$, ${Z_{g}}^{\prime }$, and ${Z_{s}}^{\prime }$ are the transposed matrices, R is a variance-covariance matrix for residual effects, and $G_{1}$ and $G_{2}$ are the variance-covariance matrix for the effect of traits. If $G_{1}$ and $G_{2}$ are approximated with $A\sigma_{a}^{2}$ and $D\sigma_{d}^{2}$ where A and D are matrices of the coefficients of coancestry among the inbred lines and the combination of F_1_ ($G_{1}=A\sigma_{a}^{2}$, $G_{2}=D\sigma_{d}^{2}$), R is described as the product of the identity matrix I and a value for the residual effect variance $\sigma_{e}^{2}$ (Sasaki 2007), Eqn. (2) was transformed as follows:


$$(\begin{array}{c} \hat{\beta }\\ {\hat{a}}_{1}\\ {\hat{a}}_{2} \end{array})={\left(\begin{array}{c} X^{\prime }X\\ {Z_{g}}^{\prime }X\\ {Z_{s}}^{\prime }X \end{array}\;\begin{array}{c} X^{\prime }Z_{g}\\ {Z_{g}}^{\prime }Z_{g}+A^{-1}\left(\sigma_{e}^{2}/\sigma_{a}^{2}\right)\\ {Z_{s}}^{\prime }Z_{g} \end{array}\;\begin{array}{c} X^{\prime }Z_{s}\\ {Z_{g}}^{\prime }Z_{s}\\ {Z_{s}}^{\prime }Z_{s}+D^{-1}\left(\sigma_{e}^{2}/\sigma_{d}^{2}\right) \end{array}\right)}^{-1}\left(\begin{array}{c} X^{\prime }y\\ {Z_{g}}^{\prime }y\\ {Z_{s}}^{\prime }y \end{array}\right)$$ (3)

A=I, and D=I were assumed. In addition, the additive genetic variances $\sigma_{a}^{2}$ and $\sigma_{e}^{2}$ were assumed to be $h^{2}\sigma_{y}^{2}$ and ${(1-h}^{2}){\;\sigma }_{y}^{2}$ using the narrow-sense heritability ($h^{2}$), respectively. Therefore, $\sigma_{e}^{2}\;/\;\sigma_{a}^{2}$ was expressed as follows:


$$\sigma_{e}^{2}/\sigma_{a}^{2}=\left(\frac{1-h^{2}}{h^{2}}\right)$$ (4)

Additionally, $\sigma_{e}^{2}/\sigma_{d}^{2}$ was calculated using the broad-sense heritability ($H^{2}$), using the same procedure.


$$\sigma_{e}^{2}/\sigma_{d}^{2}=\left(\frac{1-H^{2}}{H^{2}}\right)$$ (5)

Therefore, the normal equation was reduced to estimate the best linear unbiased estimator (BLUE) and BLUP as follows:


$$(\begin{array}{c} \hat{\beta }\\ {\hat{a}}_{1}\\ {\hat{a}}_{2} \end{array}\;)={\left(\;\begin{array}{c} X^{\prime }X\\ {Z_{g}}^{\prime }X\;\\ {Z_{s}}^{\prime }X \end{array}\;\begin{array}{c} X^{\prime }Z_{g}\\ {Z_{g}}^{\prime }Z_{g}+\;\left(\frac{1-h^{2}}{h^{2}}\right)I\\ {Z_{s}}^{\prime }Z_{g} \end{array}\;\begin{array}{c} X^{\prime }Z_{s}\\ {Z_{g}}^{\prime }Z_{s}\\ {Z_{s}}^{\prime }Z_{s}+\;\left(\frac{1-H^{2}}{H^{2}}\right)I \end{array}\;\right)}^{-1}\left(\begin{array}{c} X^{\prime }y\\ {Z_{g}}^{\prime }y\\ {Z_{s}}^{\prime }y \end{array}\right)$$ (6)

This approach does not strictly divide the variance in $H^{2}$ into components of GCA and SCA; therefore, it does not yield accurate variance estimates. However, the obtained GCAs are strongly correlated with the results of F_1_ varieties in the field inoculation test (Mitsuhashi 2024).

The results that were tested only once in the experiments were excluded from the equation to maximize the accuracy of the comparison. $H^{2}\;$was defined as the mean of the values obtained from the ANOVA for each F_1_ test plot, whereas $h^{2}$ of Pythium RSR was unknown. Therefore, based on the results of previous studies on root rot disease in maize, the ratio of narrow-sense heritability (0.51) to broad-sense heritability (0.81) for root rot discoloration (Quintas 2001) was calculated. This ratio was considered a hypothetical ratio and was multiplied by the average $H^{2}$ (0.717) to obtain the $h^{2}$ value 0.452. The program for the solution was written using R version 4.5.1 (R Core Team 2025).

### Genotyping and SNP quality control

Genomic DNA was isolated from young leaf tissue of each of the inbred lines using a standard ‘DNeasy Plant Mini Kit^TM^’ (Qiagen, Venlo, Netherlands) protocol described in our previous studies (Tamaki *et al.* 2014, 2016). Genotyping was performed using the ‘Applied Biosystems^TM^ Axiom^®^ Maize Genotyping Array 60K’ (Thermo Fisher Scientific, Massachusetts, United States), a high-density platform designed for comprehensive genomic coverage and analysis of genetic diversity in maize.

The raw genotyping data underwent rigorous quality control (QC). SNPs were filtered and removed if they exhibited a call rate < 95% or a minor allele frequency < 5%. Similarly, any inbred lines with > 10% missing genotype data were excluded from the analysis. Following this stringent QC process, a final dataset of 49,187 high-quality, polymorphic SNPs of 107 inbred lines was retained for the subsequent association analysis.

### Genome-wide association analysis

The GWAS was conducted in R version 4.5.1 using the Genomic Association and Prediction Integrated Tool (GAPIT) v3 package (Wang and Zhang 2021). To account for potential confounding owing to population structure, which can lead to spurious associations, the principal components (PCs) were calculated from the genomic marker data and included as fixed-effect covariates in all association models. The number of PCs was chosen based on genomic inflation factor (λGC), which reached ≈0.98 with the first three PCs. To ensure a robust analysis, three distinct statistical models were used and their results compared:

1. General Linear Model (GLM): A basic model that tests marker-trait associations while correcting only for population structure (PCs) as fixed covariates.

2. Mixed Linear Model (MLM): A more sophisticated model that accounts for both population structure (fixed effect) and relatedness among individuals by incorporating a genomic relationship matrix (kinship matrix) as a random effect. This model is a standard for controlling false positives in plant GWAS.

3. Bayesian-information and Linkage-disequilibrium Iteratively Nested Keyway (BLINK): An advanced multi-locus model that improves on previous methods (Huang *et al.* 2019). BLINK uses an iterative approach with two fixed-effect models. One model tests each marker while using other notably associated markers as covariates to control genetic background. The other model selects these covariates based on the Bayesian information criteria and linkage disequilibrium (LD) information. This approach enhances statistical power and computational speed, particularly for complex traits controlled by numerous loci, by eliminating the assumption that causal genes are uniformly distributed across the genome.

A genome-wide significance threshold was determined using the conservative Bonferroni correction, which is calculated as *p* < α/N, where α = 0.05 and N represents the total number of SNPs tested. This resulted in a significant threshold of *p* < 0.05/49,187, or –log_10_ (*p*) > 5.993. “Major.allele.zero = TRUE” was set, coding the major allele as 0 and the minor allele as 1. As GCA was calculated based on RSR infection frequencies, positive effect values indicated that the minor allele increased GCA toward susceptibility, whereas negative values indicated that the minor allele enhanced resistance. The kinship matrix (K) was calculated using the VanRaden method (VanRaden 2008) implemented in the GAPIT package. LD coefficient was analyzed within a ±100 kb region flanking each SNP that was determined to be significant by R version 4.5.1. A post hoc gene search ±100 kb region surrounding the significant SNP using the B73 reference genome (RefGen_v3) annotation was examined, which was also used for the genotyping array design.

## Results

### Phenotypic variation in GCA for Pythium RSR

Reflecting the characteristics of BLUP, the mean of GCAs was approximately 0 for the 202 inbred lines. However, in the subset of 107 genotyped inbred lines, variation was observed across a wide range from high resistance (negative GCA) to high susceptibility (positive GCA) (–3.119 to 10.888; mean –0.108, standard deviation 2.453, Table 2 and Fig. 2).

Two inbred lines with contrasting GCA values were identified as references for this study. The elite inbred line ‘Na71’ emerged as a key source of resistance, exhibiting a strongly favorable GCA of –2.496. Conversely, the elite inbred line ‘Ho112’ was identified as highly susceptible, with a GCA of 3.758. Both inbred lines, unlike other experimental inbred lines (e.g. ‘TI-125’ and ‘TI-113’ with superior negative GCAs), have been used for developing F_1_ commercial varieties. Furthermore, in the 2021 field inoculation test, GCA evaluations and field disease incidence of these two inbred lines were consistent (0.0% and 61.1%), although almost half of the other inbred lines in this test showed minimal to no symptoms. The differences between these two inbred lines define the range of phenotypes and provide criteria for evaluating the magnitude of the effect of the identified loci. Significant SNPs can also be verified using segregation analysis.

### BLINK model successfully identifies marker-trait associations

Association analyses using the single-locus GLM and MLM approaches did not identify any SNPs that exceeded the stringent genome-wide Bonferroni significance threshold of –log_10_ (*p*) > 5.993. The Quantile–Quantile (Q–Q) plots for these models revealed that the GLM inflated *p*-values (indicating false positives), while the MLM appeared conservative, potentially obscuring true associations (Fig. 3).

In contrast, the multi-locus BLINK model successfully identified five SNPs that were significantly associated with the GCA for Pythium RSR resistance (Table 3, Figs. 3, 4). The Q–Q plot from the BLINK analysis closely matched the expected null distribution, with noticeable deviation only for significant markers. Using the first three PCs, λGC was 0.98, indicating effective control of population structure and kinship while maintaining sufficient power to detect true marker-trait associations. Supplemental Fig. 1 shows the results of the PC analysis. With a few exceptions, the results generally followed the same trend as the classification based on our breeding origin and previous study (Mitsuhashi 2024). LD analysis revealed that no LD blocks exceeding the threshold (*r*^2^ > 0.8) were identified within ±100 kb of the five SNPs.

### Characterization of SNPs conferring enhanced resistance

Of the five SNPs that reached genome-wide significance (Table 3, Figs. 3, 4), four were associated with a positive allelic effect on GCAs, indicating that the presence of their favorable allele contributes to increased susceptibility (a higher GCA value). The remaining SNP had a negative allelic effect, suggesting that it improved resistance.

To develop practical breeding tools, this study focused on the most promising resistant-associated SNP. The SNP on chromosome 9 (AX-91781550) was associated with a GCA reduction of –0.784. This allele exhibited an effect size of –1.569 in the homozygous case, which was approximately 25.1% of the total phenotypic range (6.254) observed between the resistant ‘Na71’ and susceptible ‘Ho112’ inbred lines, highlighting its potential for achieving substantial genetic gain in breeding programs.

### Key alleles are polymorphic in elite inbred lines

The effectiveness of a molecular marker in breeding is related to its ability to differentiate between inbred lines with contrasting traits. Therefore, a critical validation procedure involved examining the allelic state of the identified SNPs in the resistant ‘Na71’ and susceptible ‘Ho112’ reference inbred lines. This analysis revealed that four of the five associated SNPs were polymorphic in these two key inbred lines. Table 4 and Supplemental Fig. 2 show the distribution of GCAs across all haplotype combinations for the five significant SNPs. Not all superior resistant inbred lines necessarily possessed AX-91781550, the sole resistant-associated SNP.

For each of the four loci, ‘Na71’ had the favorable alleles. Conversely, ‘Ho112’ had the alternate, unfavorable allele at the four loci. This finding is crucial, as it provides a direct genetic basis for the phenotypic differences between these inbred lines. This confirms that the statistically identified markers are not merely abstract associations, but rather the specific alleles that distinguish agronomically. This provides an immediate, actionable pathway for MAS in any breeding population derived from a ‘Na71 × Ho112’ (F_2_ or recombinant inbred lines).

In addition, the remaining locus (AX-108061822) was located on chromosome 3, but only five inbred lines shown in Table 4 had the unfavorable effect allele ‘G’. The effect of this allele was the largest at 2.555 (Table 3), and analyzing the segregating populations of the five inbred lines with the ‘A’ allele ‘Na71’ or ‘Ho112’ may lead to marker development. However, within ±100 kb of the most effective gene, AX-108061822, all gene models annotated in B73 RefGen_v3 were classified as low-confidence and lacked ontology, pathway, or expression annotations.

## Discussion

### Analysis of additive genetic effect related to *Pythium arrhenomanes* resistance via GCA-based GWAS

This study is the first report on the identification of specific SNP loci conferring resistance to Pythium RSR by *P. arrhenomanes* in maize through GWAS. The success of this study was primarily attributable to the use of a new methodological strategy, specifically using GCA for disease resistance as the phenotypic trait for association analysis. By calculating GCAs from multiple environment F_1_ hybrid tests, challenges such as the environmental variation that impede direct evaluation of inbred lines for resistance to soil-borne diseases, were addressed (Bernardo 2020, Griffing 1956, Mitsuhashi and Tamaki 2022). Notably, this strategy primarily targets the additive component of genetic variance, which constitutes the principal determinant of an individual’s breeding value and the primary driver of selection response (Bernardo 2020, Sprague and Tatum 1942). Consequently, our GWAS can directly identify loci that can contribute to cumulative genetic gains in F_1_ hybrid breeding programs.

### Statistical power of MLM and BLINK

The performance differences between statistical models in this study revealed the genetic structure of *Pythium arrhenomanes* resistance. The contrast between the relatively low detection power of MLM and the superior performance of BLINK (Figs. 3, 4) strongly suggests that multiple loci with minimal effects control resistance to this pathogen.

MLM reduces false positives by incorporating a genome-wide kinship matrix as a random effect; however, it may be conservative for traits with many causal variants (Kaler *et al.* 2019). Furthermore, the MLM framework incorporates genome-wide relatedness as a random effect, which may inadvertently absorb signals from loci genuinely associated with the trait. This proximity contamination can generate false negatives, particularly near causal loci, and obscure true signals (Kang *et al.* 2010).

BLINK avoids this issue by repeatedly selecting the most significant SNP as a fixed-effect covariate when testing other markers. This facilitates the evaluation of the pure effect of new markers after considering known major loci. By separating the contribution of each SNP from the global random term, BLINK recovers the statistical power lost by MLM. Therefore, the success of BLINK represents a statistical success as well as indicates that *P. arrhenomanes* resistance is distributed across multiple genomic regions. This is consistent with the resistance patterns observed against necrotrophic pathogens in diverse host–pathogen systems (Pogoda *et al.* 2021).

### Integrating resistance and susceptibility-associated markers for comprehensive MAS

Although only one SNP, AX-91781550, among the five significant loci identified by GWAS exhibited a negative effect consistent with enhanced disease resistance, the remaining SNPs with positive effect sizes may still be beneficial in breeding programs. These loci can serve as indicators of susceptibility, enabling breeders to avoid individuals carrying the effect alleles associated with increased vulnerability (Wu *et al.* 2020b). Therefore, SNPs with opposing effects can contribute to risk management strategies in MAS.

By integrating both resistance-enhancing and susceptibility-associated markers, breeders can construct a more comprehensive selection framework, improve the precision of genotype screening, and enable the development of cultivars with optimized resistance profiles. This dual approach enhances prediction accuracy and accelerates breeding progress in various crops (Zeng and Kang 2025).

AX-108061822 exhibited the largest allelic effect but had an extremely low minor allele frequency (Table 3). Although this pronounced effect is notable, the rarity of the allele raises concerns about potential bias from a small number of disproportionately affected inbred lines and instability due to its low frequency (Yang *et al.* 2014). LD analysis within a ±100 kb region revealed no contiguous block meeting the *r*^2^ threshold of 0.8 (Tardivel *et al.* 2019), and no annotated genes with known functions were identified in this interval. These findings suggest that the SNP may directly tag a causative polymorphism acting as a surrogate marker within a larger haplotype block (Atwell *et al.* 2010). To assess its predictive utility, validation experiments will be performed using segregating populations derived from inbred lines carrying or lacking the effect allele (Table 4). Further fine-mapping or transcriptome-based validation will be required in the future.

### Acceleration of the breeding cycle

Traditional breeding for quantitative disease resistance is time-consuming and resource-intensive because it relies on phenotypic selection in field trials over several generations, and the development and release of elite inbred lines typically requires an additional 10 years. The markers identified in this study enable MAS during the seedling stage (Abdulmalik *et al.* 2017). Breeders can extract DNA from small leaf fragments of each seedling in a segregating population (the F_2_ generation of ‘Na71 × Ho112’) and use simple and rapid genotyping methods such as Kompetitive allele specific PCR (KASP) to identify individuals that have inherited the desirable resistance allele (Chen *et al.* 2021) from ‘Na71’. Furthermore, susceptible individuals can be eliminated within a few weeks after germination, enabling breeders to concentrate resources on genetically superior plants. This approach substantially reduces the selection cycle, increases the annual rate of genetic progress, and facilitates the rapid delivery of improved varieties to farmers (Gedil and Menkir 2019).

### Conclusion and future perspectives

In conclusion, this study successfully identified new agronomically significant loci associated with resistance to Pythium RSR caused by *P. arrhenomanes* using GCA-based GWAS. This study demonstrated that these identified SNPs are readily applicable to MAS, providing an effective tool to accelerate the breeding of resistant maize varieties.

Of the five significant SNPs detected in this study, only one showed a resistance effect, whereas the remaining four tended to increase susceptibility to disease. This combination of positive and negative effects is characteristic of polygenic traits and suggests that resistance results from the cumulative influence of numerous small-effect factors (Wu *et al.* 2025). However, these five SNPs might not explain all phenotypes (Table 4). Other factors, such as undetected resistance factors, structural variations (Gui *et al.* 2022), or gene-gene interactions (Durand *et al.* 2012), may also contribute. Future studies should analyze the abovementioned segregating population, elucidate the remaining genetic variance through measures such as expanding sample sizes, conducting meta-analyses (Ali *et al.* 2013), identifying rare variants through whole-genome sequencing, and verifying the functions of candidate genes using techniques such as transcript expression analysis (qRT-PCR) in resistant and susceptible inbred lines following pathogen exposure, and reverse genetics approaches such as virus-induced gene silencing (Hou *et al.* 2023) or CRISPR/Cas9-mediated knockout (Gentzel *et al.* 2020). The identification of causal genes will provide markers for breeding as well as reveal fundamental insights into the molecular mechanisms of the maize immune response to *Pythium* pathogens, thereby facilitating new strategies for engineering durable disease resistance.

## Author Contribution Statement

S.M. consistently designed and conducted the experiments, sample preparation, data analysis, and authored this manuscript.

## Supplementary Material

Supplemental Figures

## Figures and Tables

**Fig. 1.**
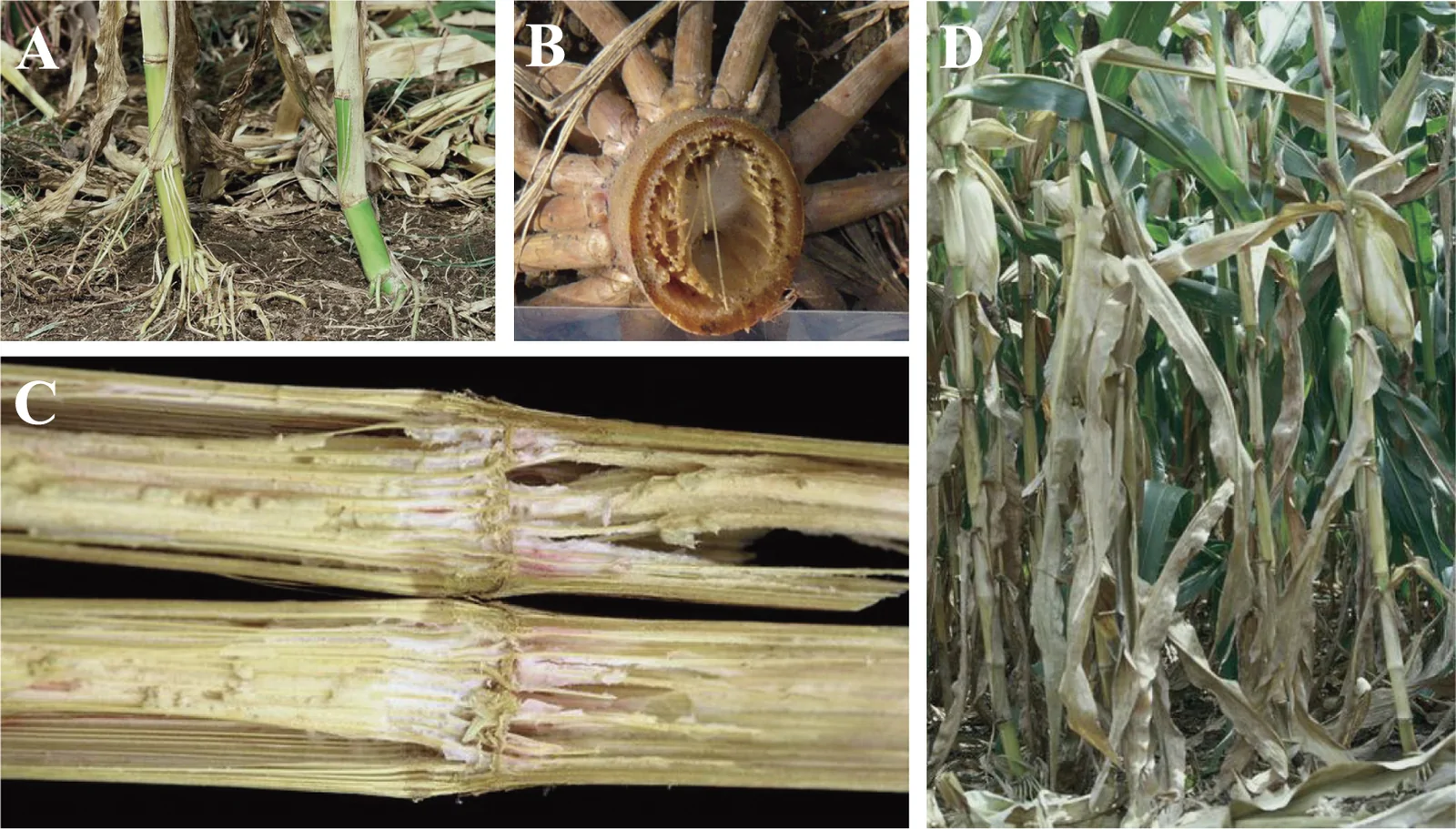
Typical symptoms of Pythium root and stalk rot (RSR) caused by *Pythium arrhenomanes*. A: Healthy (right) and diseased plants (left), B: Symptoms of root and bottom of the stalk rot, C: Symptoms of stalk rot, D: Symptoms of drooping ears in the field affected by Pythium RSR.

**Fig. 2.**
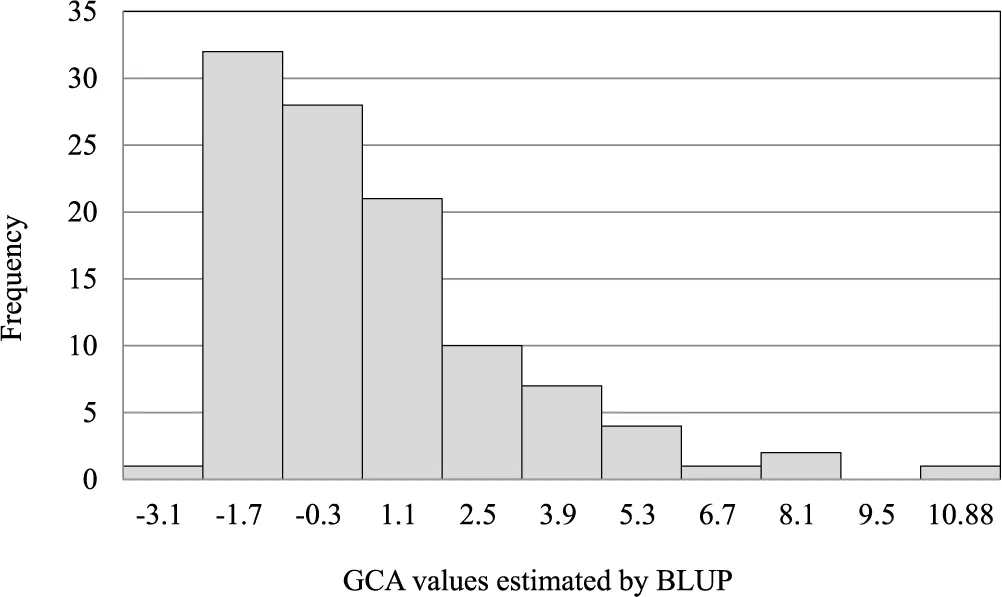
Histogram of general combining ability (GCA) values derived from best linear unbiased prediction (BLUP).

**Fig. 3.**
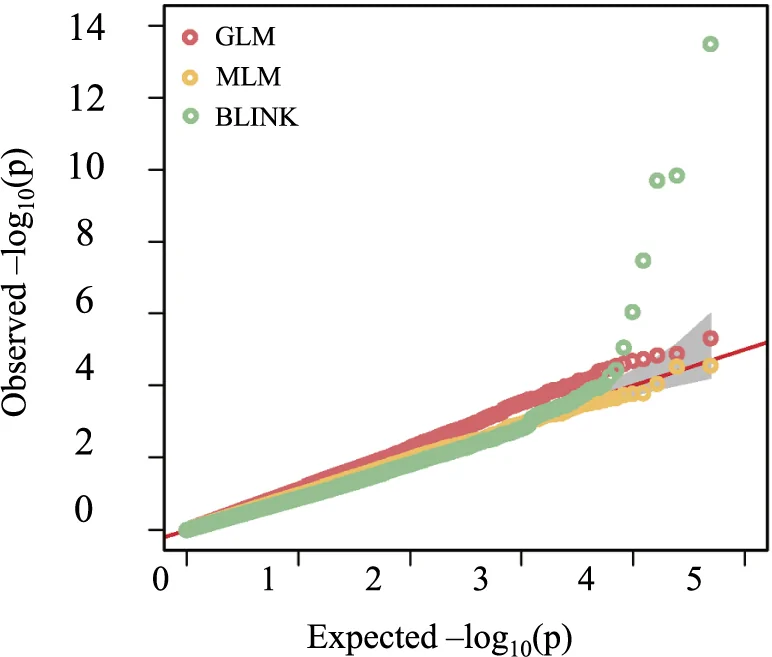
Quantile–Quantile plot of SNPs significantly associated with Pythium root and stalk rot resistance using different models. GLM, General Linear Model; MLM, Mixed Linear Model; BLINK, Bayesian-information and Linkage-disequilibrium Iteratively Nested Keyway.

**Fig. 4.**
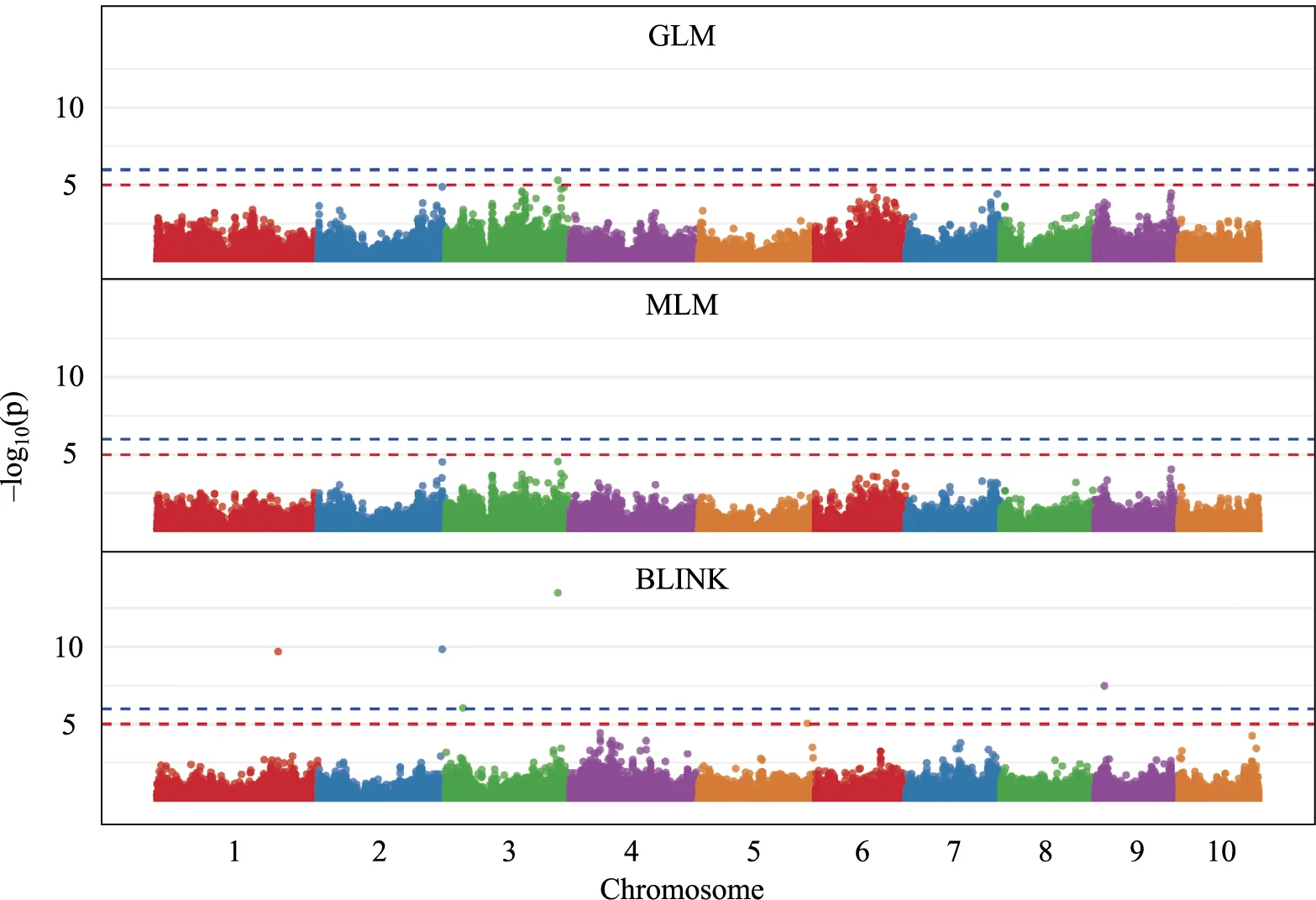
Manhattan plots of SNPs significantly associated with Pythium root and stalk rot resistance using different models. GLM, General Linear Model; MLM, Mixed Linear Model; BLINK, Bayesian-information and Linkage-disequilibrium Iteratively Nested Keyway. Red dotted line: –log_10_ (*p*) = 5.000. Blue dotted line: –log_10_ (*p*) = 5.993.

**Table 1.** Scale and outline of field test, which tested 946 F_1_ data derived from 202 inbred lines through natural infection from 2014 to 2024

Field experiment number	Number of				Broad-sense heritability ($H^{2}$)
	Hybrids	Inbred lines crossed for hybrids	Plants in a single row	Replications	
2014-1	39	29	19	2	0.908
2014-2	19	30	19	3	0.924
2015-1	34	29	19	2	0.789
2015-2	16	22	19	3	0.815
2016-1	14	14	19	4	0.970
2016-2	7	5	19	3	0.050^†^
2016-3	23	22	19	2	0.651
2016-4	47	50	13	2	0.517
2016-5	36	34	13	2	0.562
2017-1	22	17	19	2	0.824
2017-2	18	15	19	2	0.840
2017-3	80	42	13	2	0.082^†^
2017-4	37	28	13	2	0.204
2017-5	13	19	19	3	0.867
2018-1	7	12	19	4	0.278
2018-2	28	3	19	2	0.749
2018-3	52	36	13	2	0.746
2018-4	26	32	13	2	0.652
2018-5	8	15	19	3	0.915
2019-1	11	16	19	4	0.796
2019-2	17	24	19	2	0.668
2019-3	20	22	13	2	0.935
2019-4	10	13	13	2	0.845
2019-5	11	17	19	3	0.697
2020-1	3	5	19	4	0.969
2020-2	100	49	13	2	0.083^†^
2020-3	4	8	19	3	0.345
2020-4	2	4	19	3	0.988
2021-1	77	62	13	2	0.022^†^
2023-1	38	40	19	2	0.673
2023-2	31	32	13	2	0.075^†^
2024-1	67	56	13	2	0.674
2024-2	25	26	19	2	0.275
2024-3	4	8	19	3	0.000^†^

Sowing and observation dates were different for each year or environment at the ILGS, NARO, Nasushiobara, Tochigi, Japan. A total of 724 F_1_ hybrids were tested, obtaining 946 data. These F_1_ hybrids were derived from crosses among 202 inbred lines. ^†^ These were excluded from the calculation of the mean as exaggerated values (Smirnov-Grubbs test *p* < 0.05). The average value of $H^{2}$ was 0.717.

**Table 2.** General combining abilities (GCAs) for Pythium root and stalk rot in the 107 inbred lines maize diversity panel

Inbred name	Group^†^	GCAs	Developed by^‡^		Inbred name	Group^†^	GCAs	Developed by^‡^
Mi29	D	5.580	KOARC, NARO		N19-D05	D	1.333	ILGS, NARO
Mi29SRR	D	10.888	KOARC, NARO		N19-F01	JF	–2.185	ILGS, NARO
Mi47	JF	1.543	KOARC, NARO		N19-F03	JF	–2.219	ILGS, NARO
Mi62	RD	0.740	KOARC, NARO		N20-D01	D	–1.755	ILGS, NARO
Mi88	D	–1.837	KOARC, NARO		N20-D02	D	–2.070	ILGS, NARO
Mi91	RD	0.169	KOARC, NARO		N21-D01	D	–1.104	ILGS, NARO
Mi103	JF	1.282	KOARC, NARO		N21-D02	D	0.678	ILGS, NARO
Mi108	D	3.457	KOARC, NARO		N21-D04	D	–1.371	ILGS, NARO
Mi109	D	5.196	KOARC, NARO		N21-D05	D	–2.555	ILGS, NARO
Mi110	D	2.566	KOARC, NARO		N21-D06	D	–2.512	ILGS, NARO
Mi111	JF	–1.894	KOARC, NARO		N21-F01	JF	–0.892	ILGS, NARO
Mi113	RD	0.617	KOARC, NARO		Na50	JF	7.247	ILGS, NARO
Mi114	JF	3.916	KOARC, NARO		Na65	D	1.414	ILGS, NARO
Mi115	JF	–1.936	KOARC, NARO		Na71	D	–2.496	ILGS, NARO
N09-07	JF	–2.927	ILGS, NARO		Na83	JF	3.512	ILGS, NARO
N10-01	D	–1.073	ILGS, NARO		Na91	JF	0.085	ILGS, NARO
N10-02	D	–0.927	ILGS, NARO		Na98	D	–2.514	ILGS, NARO
N10-08	JF	–0.524	ILGS, NARO		Na100	D	2.651	ILGS, NARO
N10-12	JF	–0.699	ILGS, NARO		Na102	D	–2.277	ILGS, NARO
N11-02	D	–1.345	ILGS, NARO		Na105	JF	–1.628	ILGS, NARO
N12-01	D	–2.085	ILGS, NARO		Na106	JF	0.258	ILGS, NARO
N12-02	JF	–0.756	ILGS, NARO		Na107	JF	6.879	ILGS, NARO
N12-05	JF	–1.293	ILGS, NARO		Na108	JF	–1.252	ILGS, NARO
N12-06	JF	0.279	ILGS, NARO		Na109	D	–1.163	ILGS, NARO
N12-07	JF	–1.808	ILGS, NARO		Na111	JF	–0.728	ILGS, NARO
N12-08	JF	0.147	ILGS, NARO		Na112	MIS (JF)	0.174	ILGS, NARO
N13-01	D	–1.728	ILGS, NARO		Ho95	JF	–2.800	HARC, NARO
N13-02	JF	–1.171	ILGS, NARO		Ho102	D	–0.576	HARC, NARO
N13-03	JF	–1.332	ILGS, NARO		Ho108	D	4.492	HARC, NARO
N13-04	JF	3.702	ILGS, NARO		Ho110	D	0.265	HARC, NARO
N13-05	JF	0.111	ILGS, NARO		Ho112	D	3.758	HARC, NARO
N13-06	JF	1.618	ILGS, NARO		Ho123	D	–0.459	HARC, NARO
N13-07	JF	–1.206	ILGS, NARO		Ho124	NEF	–2.036	HARC, NARO
N13-08	JF	–1.793	ILGS, NARO		Ho125	JF	1.028	HARC, NARO
N14-01	D	–0.523	ILGS, NARO		Ho133	D	–2.366	HARC, NARO
N14-02	D	–1.929	ILGS, NARO		TI-064	NEF	–1.090	HARC, NARO
N14-03	D	–1.471	ILGS, NARO		TI-089	D	0.316	HARC, NARO
N15-01	D	–2.751	ILGS, NARO		TI-113	NEF	–2.981	HARC, NARO
N16-03	D	1.397	ILGS, NARO		TI-125	D	–3.119	HARC, NARO
N16-04	D	–1.877	ILGS, NARO		TI-133	MIS (D*EF)	1.823	HARC, NARO
N16-07	JF	–0.159	ILGS, NARO		TI-134	D	0.213	HARC, NARO
N17-D01	D	–2.645	ILGS, NARO		TI-135	D	–0.210	HARC, NARO
N17-D02	D	0.115	ILGS, NARO		TI-137	NEF	–0.293	HARC, NARO
N17-D03	D	–1.409	ILGS, NARO		TI-140	D	–1.839	HARC, NARO
N17-D04	D	–2.495	ILGS, NARO		TI-142	NEF	–2.163	HARC, NARO
N17-F01	JF	1.565	ILGS, NARO		TI-144	NEF	–2.532	HARC, NARO
N17-F02	JF	1.346	ILGS, NARO		TI-147	D	4.816	HARC, NARO
N17-F03	JF	–1.906	ILGS, NARO		TI-148	D	–0.974	HARC, NARO
N18-D01	D	1.341	ILGS, NARO		TI-161	D	–1.083	HARC, NARO
N18-D02	D	2.751	ILGS, NARO		CHU44	JF	–0.310	CAES, Nagano pref.
N18-F01	JF	–2.327	ILGS, NARO		CHU68	JF	0.154	CAES, Nagano pref.
N18-F02	JF	0.347	ILGS, NARO		KK109	D	–0.920	GAFSA
N19-D01	D	–2.135	ILGS, NARO		KK153	JF	0.477	GAFSA
N19-D02	D	–1.317	ILGS, NARO					

The BLUP-derived GCA of 107 inbred lines ranged from –3.119 to 10.888 (Mean –0.108, standard deviation 2.453). ^†^ D, dent; JF, Japanese flint; EF, European flint; NEF, Northern European flint; RD, Mainly developed from hybrids for summer seeding; MIS, Miscellaneous origin. ^‡^ KARC, NARO: Kyushu Okinawa Agricultural Research Center, NARO. IRGS, NARO: Institute of Livestock and Grassland Science, NARO. HARC, NARO: Hokkaido Agricultural Research Center, NARO. CAES, Nagano pref.: Chushin Agricultural Experiment Station, Nagano Prefecture. GAFSA: Japan Grassland Agriculture & Forage Seed Association.

**Table 3.** Significant single-nucleotide polymorphisms (SNPs) associated with general combining ability (GCA) for Pythium root and stalk rot resistance identified by the Bayesian-information and Linkage-disequilibrium Iteratively Nested Keyway model

No.	SNPs ID	Chromosome	Position (bp)	*p*-value	Allelic effect (GCA)	Major allele	Minor (effective) allele and frequency	
1	AX-107954516	1	226,610,900	2.02 × 10^–10^	1.022	A	C	0.257
2	AX-108013530	2	232,030,735	1.46 × 10^–10^	1.219	G	A	0.243
3	AX-108052057	3	133,165,458	9.01 × 10^–7^	0.700	T	C	0.491
4	AX-108061822	3	210,314,266	3.22 × 10^–14^	2.555	A	G	0.051
5	AX-91781550	9	118,250,981	3.36 × 10^–8^	–0.784	C	T	0.491

**Table 4.** Genotypes of the associated SNPs associated with Pythium root and stalk rot resistance in ‘Na71’ (resistant), ‘Ho112’ (susceptible) and other inbred lines

Name	Group	GCAs	No./Genotype or effect at each SNP				
			1	2	3	4	5
Na71	D	–2.496	A	G	T	A	T
Ho112	D	3.758	C	A	C	A	C
TI-125	D	–3.119	A	G	T	A	T
TI-113	NEF	–2.981	A	G	Y	R	C
N09-07	JF	–2.927	A	G	T	A	T
Ho95	JF	–2.800	C	G	T	A	C
N15-01	D	–2.751	C	G	T	A	T
N17-D01	D	–2.645	C	G	T	A	T
N21-D05	D	–2.555	A	G	T	A	T
TI-144	NEF	–2.532	A	G	T	A	C
Na98	D	–2.514	A	G	C	A	T
N21-D06	D	–2.512	A	G	T	A	T
Mi29SRR	D	10.888	A	A	C	G	C
Na50	JF	7.247	A	A	C	G	C
Na107	JF	6.879	A	G	C	G	C
Na83	JF	3.512	A	G	C	G	T
Na109	D	–1.163	A	G	N	G	T
Minor (effect) allele			C	A	C	G	T
Allelic effect			1.022	1.219	0.700	2.555	–0.784

D, dent; JF, Japanese flint; NEF, Northern European flint. Ambiguous nucleotide codes follow the IUPAC standard: R (A/G) and Y (C/T). In this dataset, these indicate heterozygous genotypes (0/1). N indicates undetermined nucleotide.
