# Supplementary material for: Genome-wide association study identifies loci for the general combining ability of Pythium root and stalk rot caused by Pythium arrhenomanes in maize (Zea mays L.)
Source: Breed Sci. 2026 Jun 16;76(3):240–50. doi: 10.1270/jsbbs.25069 (PMC13709631; doi:10.1270/jsbbs.25069)
Supplement: Supplementary file 1 [file 76_240_s1.pdf]

doi: 10.1270/jsbbs.25069

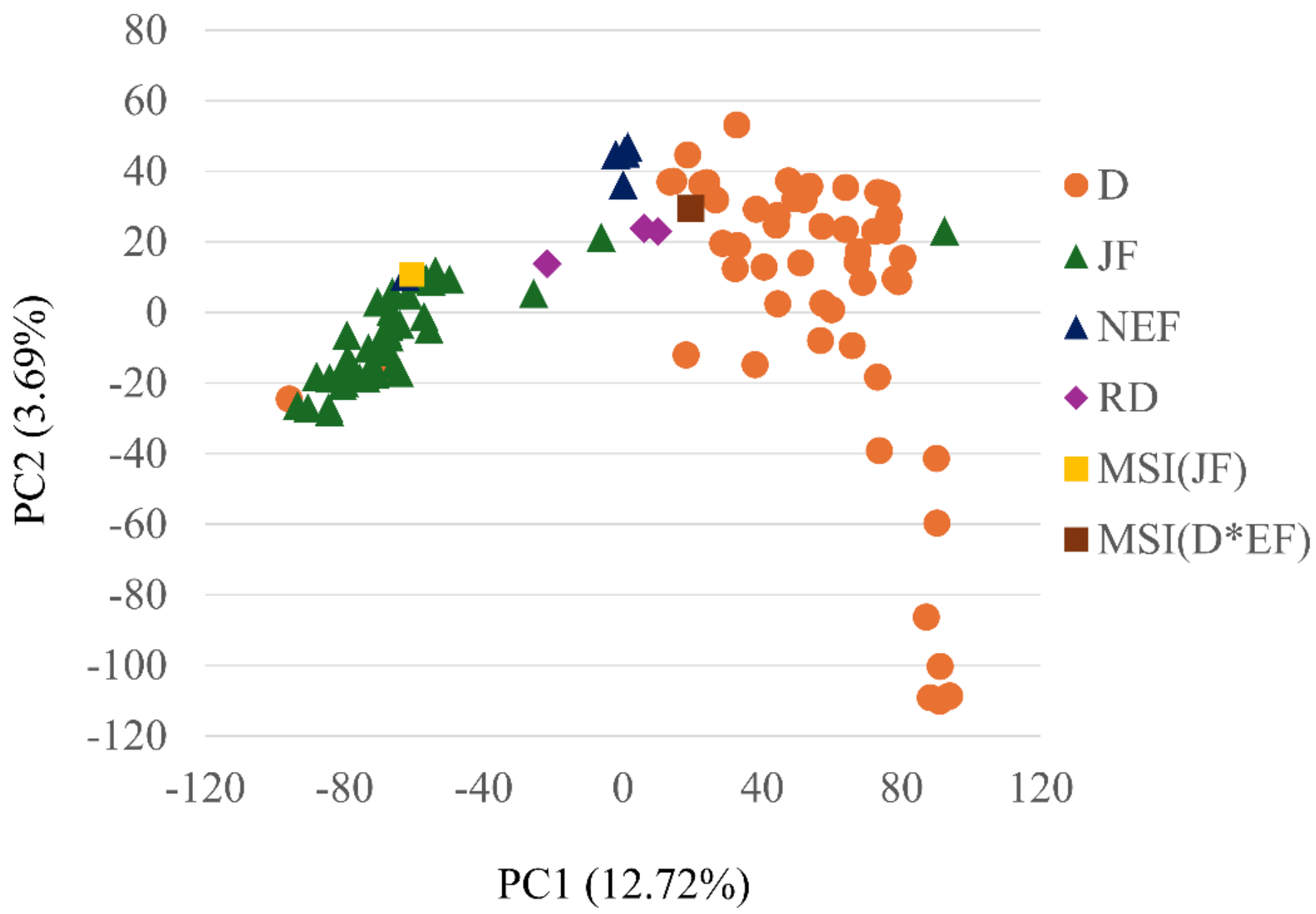

Supplemental Fig. 1. Plot of principal component (PC) 1 and PC 2 scores based on 49,187 SNP markers of the 107 inbred lines. D, dent; JF, Japanese flint; EF, European flint; NEF, Northern European flint; RD, Mainly developed from hybrids for summer seeding; MIS, Miscellaneous origin.

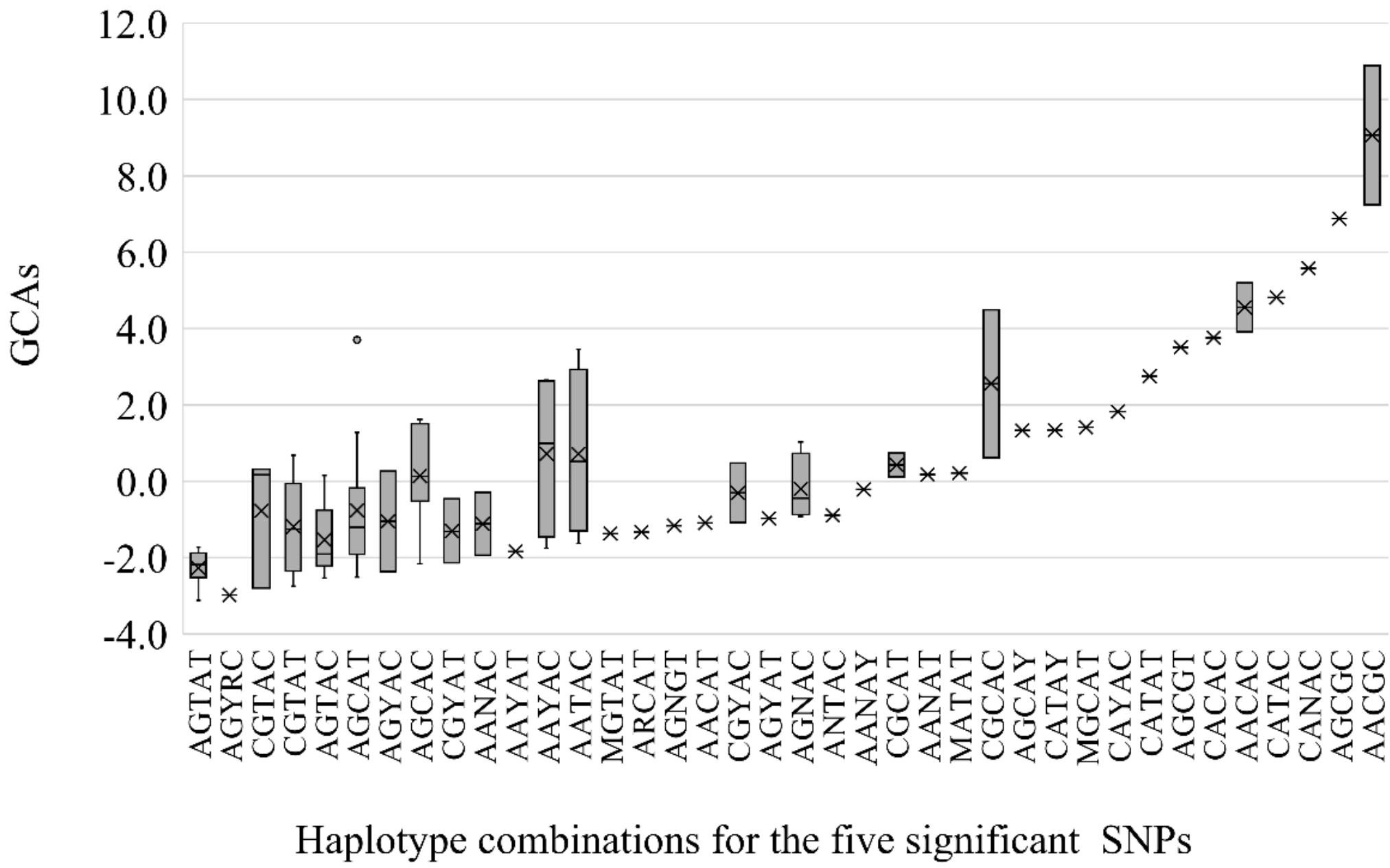

Supplemental Fig. 2. Distribution of general combining abilities (GCAs) across haplotype combinations for the five significant SNPs identified by genome-wide association study. Ambiguous nucleotide codes follow the IUPAC standard: R (A/G), Y (C/T), M (A/C), and N (any base). In this dataset, R, Y, and M indicate heterozygous genotypes (0/1), whereas N represents an undetermined nucleotide (missing or ambiguous call [./.]).
